# Presence of T cells directed against CD20-derived peptides in healthy individuals and lymphoma patients

**DOI:** 10.1007/s00262-019-02389-7

**Published:** 2019-09-07

**Authors:** Benoit Milcent, Nathalie Josseaume, Quentin Riller, Ilenia Giglioli, Emilia Rabia, Claire Deligne, Jean-Baptiste Latouche, Mohamad Hamieh, Alexandre Couture, Olivier Toutirais, Yu-Chun Lone, Raphaël Jeger-Madiot, Stéphanie Graff-Dubois, Sandy Amorim, Pascale Loiseau, Antoine Toubert, Pauline Brice, Catherine Thieblemont, Jean-Luc Teillaud, Sophie Sibéril

**Affiliations:** 1 Sorbonne Université, Sorbonne Paris Cité, Université Paris Descartes, Université Paris Diderot, Inserm UMRS 1138, “Cancer, Immune Control and Escape” Laboratory, Centre de Recherche des Cordeliers, Paris, France; 2 Inserm U1245, Institute for Research and Innovation in Biomedicine (IRIB), Normandie University, Rouen University Hospital, Rouen, France; 3 Unicaen, Inserm 1237, Physiopathology and Imaging of Neurological Disorders, Normandie University, Caen, France; 4 French Blood Service (Etablissement Français du Sang, EFS), Caen, France; 5 Inserm U1014, Hôpital Paul Brousse, Villejuif, France; 6 Inserm U1135, CNRS ERL8255, Center for Immunology and Microbial Infection, Paris, France; 7 APHP, Saint-Louis Hospital, Hemato-oncology, Diderot University, Sorbonne Paris Cité, Paris, France; 8 Laboratoire d’Immunologie et Histocompatibilité, Hôpital Saint-Louis, Paris, France; 9 Inserm UMR-S 1160, Paris, France; 10 Institut Universitaire d’Hématologie, Université Paris Diderot-Paris 7, Paris, France; 11 EA7324 Université Paris Descartes, Sorbonne Paris Cité, Paris, France; 12 Present Address: Laboratory “Immune Microenvironment and Immunotherapy”, Sorbonne Université UMRS 1135, INSERM U.1135, Centre d’Immunologie et des Maladies Infectieuses (CIMI), Paris, France; 13 Cordeliers Research Center-Inserm UMR-S 1138, “Cancer, Immune Control and Escape” Laboratory, 15 rue de l’Ecole de Médecine, 75006 Paris, France

**Keywords:** CD4^+^ T cell responses, Follicular lymphoma, Human CD20-derived peptides, Non-mutated self-peptides

## Abstract

Preclinical and clinical studies have suggested that cancer treatment with antitumor antibodies induces a specific adaptive T cell response. A central role in this process has been attributed to CD4^+^ T cells, but the relevant T cell epitopes, mostly derived from non-mutated self-antigens, are largely unknown. In this study, we have characterized human CD20-derived epitopes restricted by HLA-DR1, HLA-DR3, HLA-DR4, and HLA-DR7, and investigated whether T cell responses directed against CD20-derived peptides can be elicited in human HLA-DR-transgenic mice and human samples. Based on in vitro binding assays to recombinant human MHC II molecules and on in vivo immunization assays in H-2 KO/HLA-A2^+^-DR1^+^ transgenic mice, we have identified 21 MHC II-restricted long peptides derived from intracellular, membrane, or extracellular domains of the human non-mutated CD20 protein that trigger in vitro IFN-γ production by PBMCs and splenocytes from healthy individuals and by PBMCs from follicular lymphoma patients. These CD20-derived MHC II-restricted peptides could serve as a therapeutic tool for improving and/or monitoring anti-CD20 T cell activity in patients treated with rituximab or other anti-CD20 antibodies.

**Electronic supplementary material:**

The online version of this article (10.1007/s00262-019-02389-7) contains supplementary material, which is available to authorized users.

## Introduction

A number of anti-CD20 therapeutic antibodies are now successfully used to treat B cell lymphomas and CLL [1, 2]. The CD20 membrane-spanning 4A molecule is an unglycosylated phosphoprotein (33–37 kDa, 297 amino acids) encoded by the *MS4A1* gene and expressed by B cells from the early pre-B cell to the late B cell stages. Pro-B cells do not express CD20. CD20 disappears when B cells differentiate into plasma cells [3–5]. CD20 is involved in the regulation of intracellular calcium levels and in B cell signaling, proliferation, and differentiation [6–9]. It contains two extracellular loops—one small and one large—containing the epitopes bound by anti-CD20 antibodies [10, 11].

We and others have shown in a mouse model that CD4^+^ T cells play a critical role in the long-term antitumor protection elicited by anti-CD20 treatment [12–14]. T cell depletion and T cell transfer experiments demonstrated that anti-CD20 treatment leads to the development of a potent and specific memory CD4^+^ T cell response against CD20^+^ tumor cells [12, 14]. Another study showed that anti-CD20 mAb engages FcγRIIA expressed on dendritic cells leading to the priming of self-reactive tumor-specific CD4^+^ T cells [14]. However, the specific T cell epitopes involved in this process are unknown.

Analyses of the HLA ligandome in healthy donors or patients with B cell malignancies have allowed the identification of self-peptides derived from B cell molecules, in particular CD19 and CD20, that could be recognized by T cells [15, 16]. Immunogenic MHC I-restricted CD20-derived peptides have also been identified in studies using an in silico approach and in vitro assays based on stimulation of CTLs with candidate peptides [17–21]. Notably, one particular highly immunogenic peptide located in the CD20 transmembrane domain and recognized by CD8^+^ T cells, CD20_188–196_ (SLFLGILSV), induces the expansion of CTLs in healthy donors and patients. These cells efficiently kill primary tumor cells or cells from cell lines derived from B cell malignancies [17–21]. A strategy developed to detect and expand allo-MHC-restricted T cells reactive to self-tumor antigens has also resulted in the characterization of 20 non-mutated HLA-A*02:01-restricted epitopes from CD20 [22]. However, these studies have been largely focused on MHC I-restricted CD20 epitopes. Only one study has reported that a CD20 alternative splicing isoform expressed in patients with B cell lymphoma can generate immunogenic CD4^+^ T cell epitopes [23]. Thus, the identification of MHC II-restricted peptides derived from native non-mutated CD20 molecule is still needed to better understand the role of CD4^+^ T cells in the long-term response to anti-CD20 treatment.

In this study, we assessed whether human CD20-derived MHC II-restricted immunogenic peptides can be identified using a combination of in vitro binding assays to recombinant human MHC II molecules and subsequent in vivo immunization experiments in human HLA-DR-transgenic mice. We could identify a number of CD20-derived MHC II-restricted long peptides (*n *= 21) localized in the extracellular, transmembrane and intracellular domains of CD20. These peptides induce in vitro IFN-γ responses in PBMCs from healthy donors (HD) and follicular lymphoma (FL) patients.

## Materials and methods

### Human samples

PBMCs from HD were obtained from Cellular Technology Limited (CTL)—Europe (*n* = 25) or from the French blood agency (Etablissement Français du Sang, EFS) (*n *= 11). HD from CTL—Europe were selected as expressing at least one of the following HLA-DR alleles: HLA-DRB1*01:01, HLA-DRB1*03:01, HLA-DRB1*04:01, and HLA-DRB1*07:01. Anonymous HD from EFS were not HLA-typed. PBMCs were also obtained from patients diagnosed with high tumor-burden follicular lymphoma (FL; *n *= 9) and treated with a regimen consisting of rituximab combined with chemotherapy (cyclophosphamide, doxorubicin, vincristine, prednisolone) (R-CHOP) (Hemato-oncology Department, Saint Louis hospital, Paris, France). Blood samples from patients were collected 6 weeks after initiation of treatment. HLA typing of patients was performed using the Polymerase Chain Reaction-Sequence Specific Oligoprobe (PCR-SSO) molecular method using the LABType SSO kits from One Lambda Inc. (Canoga). Spleens (*n *= 7) were obtained from organ transplant donors at the Hôpital Pitié-Salpêtrière (Paris, France).

### Mice

For immunization, 8- to 12-week-old HLA-A2.1-/HLA-DR1-transgenic H-2 class I-/class II-knockout (KO) female mice were used [24].

### Definition of MHC II-restricted human CD20-derived candidate peptides

Bioinformatics tools for epitope prediction (SYFPEITHI; IEDB; BIMAS) applied to the human CD20 sequence deposited in the NCBI database (NP_068769.2) were used to identify MHC II-restricted CD20-derived candidate peptides. The candidate peptides were then screened at ProImmune for high MHC-peptide binding scores with the high-throughput ProImmune REVEAL^®^ MHC-peptide binding assay (https://www.proimmune.com/ecommerce/page.php?page = reveal_class2). This binding assay quantifies the ability of test peptides to bind to the human MHC II molecules HLA-DR1 (allele HLA-DRB1*01:01), HLA-DR3 (allele HLA-DRB1*03:01), HLA-DR4 (allele HLA-DRB1*04:01), and HLA-DR7 (allele HLA-DRB1*07:01), and also measures the ability of the bound peptide to stabilize the resulting MHC II-peptide complex. The assay is based on determining the presence or absence of the native conformation of the MHC II-peptide complex, as recognized by a specific antibody. Each peptide is given a score relative to a positive control peptide, which is known to bind MHC II molecules with high affinity.

### Immunization of mice

Ten 8- to 12-week-old HLA-A2.1-/HLA-DR1-transgenic female mice (HLA-DRB1*01:01) were intravenously injected with 2 × 10^5^ EL4 mouse thymoma cells expressing human CD20 (EL4-huCD20) [25]. Splenic CD4^+^ T cells from these immunized mice were isolated 21 days after injection using the CD4^+^ T cell negative selection kit (Miltenyi Biotec) and pooled. The purified pooled CD4^+^ T cells (10^5^ cells/well) were then incubated in ELISPOT plates for 36 h with 10^4^ HLA-DRB1*01:01-expressing artificial antigen-presenting cells (AAPCs) derived from NIH-3T3 cells [26] which were loaded with the CD20-derived peptide mixture.

### ELISPOT assays

Human PBMCs (*n *= 26 for HD and *n *= 9 for FL patients) or spleen cells (*n *= 7) (2 × 10^6^ cells/ml) were cultured for 7 days in RPMI1640 medium supplemented with 10% heat-inactivated human serum, 1% l-glutamine, 1% penicillin, and streptomycin (Life Technologies) in the presence of CD20-derived peptide mixture (10 µg of each peptide). A pool of two human MHC II-restricted Factor VIII (FVIII)-derived peptides (peptide 1972, KMALYNLYPGVFETV, and peptide 2145, IARYIRLHPTHYSIR) was used as a control of self-antigen-derived peptides that bind to human HLA-DR molecules [27–29]. In some experiments, 10 μg/ml of blocking anti-HLA-DR, DP, DQ monoclonal antibody (clone Tu39; BD Biosciences) were added to the culture. On day 1, 50 ng/ml IL-7 (Peprotech) and on day 3, 50 UI/ml IL-2 (Peprotech) were added to the culture. On day 7, 10^5^ PBMCs or splenocytes/well were incubated in IFN-γ ELISPOT plates (CTL—Europe) for 36 h in serum-free medium with FVIII- or CD20-derived peptides, in the presence or absence of anti-HLA-DR, -DP, -DQ blocking antibody. Each sample was tested in triplicate and the mean value of each triplicate was reported. The positive threshold was set at ≥ 10 SFU per 10^5^ cells after subtracting the background noise, as described previously [30]. In other experiments, 5 × 10^5^ PBMCs/well from HD (*n *= 10) were tested ex vivo in the absence of cytokines with either the peptide pool (containing 10 µg of each peptide) or with each of the 21 peptides (10 µg) (1 well/peptide or peptide pool). Human and mouse IFN-γ ELISPOT assays were performed with the Single Color ELISPOT kit according to the manufacturer’s recommendations (CTL—Europe). Following completion of the ELISPOT protocol, the plates were air dried in a laminar flow hood prior to analysis. The resulting spots were counted using a computer-assisted ELISPOT image analyzer (S6 Ultra-V Analyzer, CTL-Europe) customized for analyzing ELISPOT assays to meet the objective criteria for size, chromatic density, shape, and color. Spot forming units (SFU) were automatically calculated by the Immunospot SC Suite Software (CTL—Europe) using the SmartCount™ and Autogate™ functions.

### Statistical analyses

Statistical evaluation of mouse and human ELISPOT data was performed using non-parametric paired (Wilcoxon) or unpaired (Mann–Whitney) tests, and multiple *t* tests with Bonferroni correction (indicated in each figure legend). Prism software (version 5, Graphpad, San Diego, CA, USA) was used for statistical analyses. For all statistical tests performed, *p* values were considered significant if ≤ 0.05.

## Results

### CD20-derived peptides that bind strongly to human MHC II are immunogenic in HLA-DR transgenic mice

**Fig. 1:**
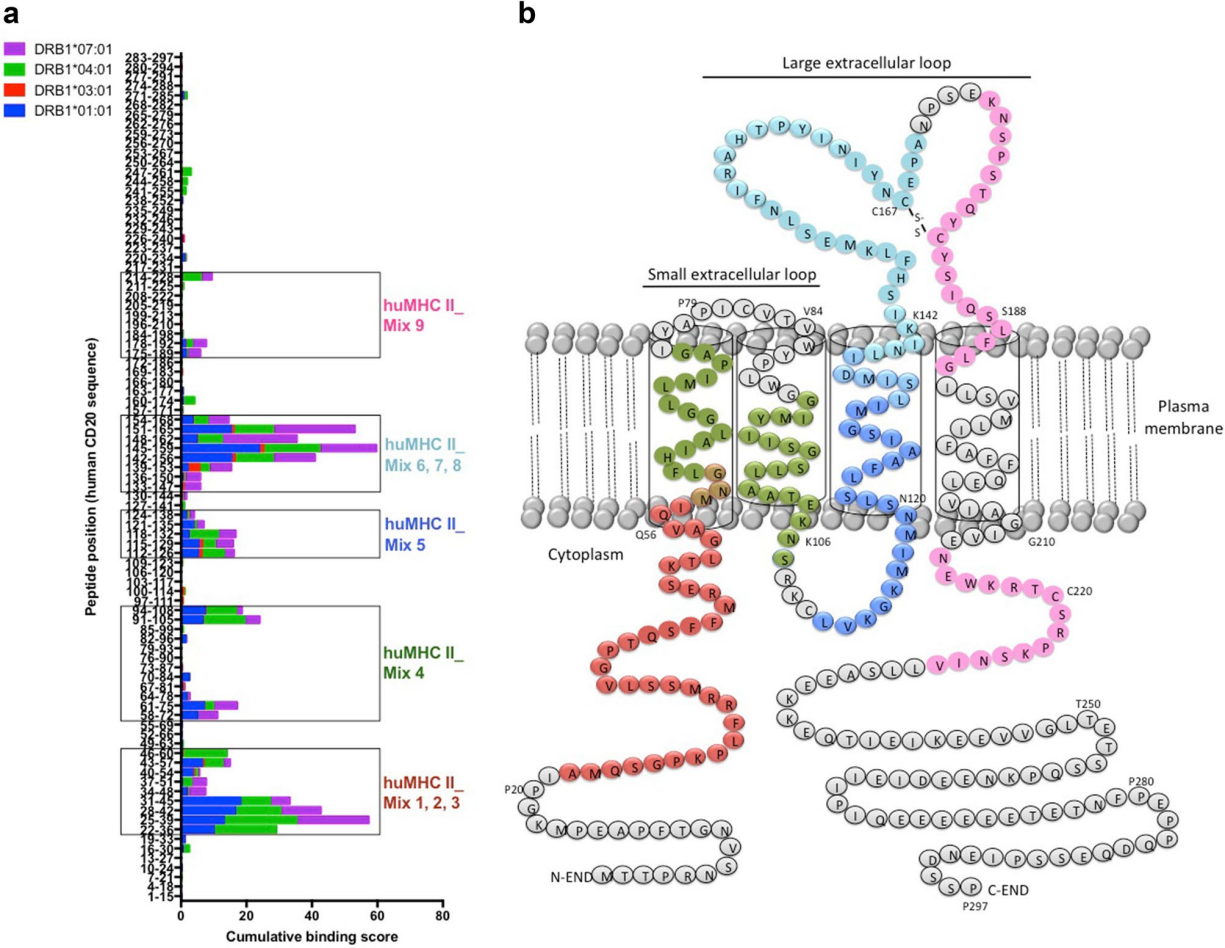
Screening of immunogenic HLA-DR-restricted CD20-derived peptides. **a** Cumulative scores of the binding of human CD20-derived peptides to recombinant HLA-DRB1*01:01 (blue), *03:01 (red), *04:01 (green), and *07:01 (purple) molecules as calculated with the ProImmune REVEAL^®^ MHC-peptide binding assay. High scoring peptides within intracellular, transmembrane, and extracellular domains of the human CD20 molecule were pooled into 9 different mixtures of 18 to 20-mer MHC II-restricted peptides (huMHC II_Mix 1 to 9) (see also Supplementary Table 1). **b** Localization of the different MHC II-restricted CD20-derived peptide mixtures (huMHC II_Mix 1 to 3 in red; huMHC II_Mix 4 in green; huMHC II_Mix 5 in dark blue; huMHC II_Mix 6 to 8 in light blue; huMHC II_Mix 9 in pink)

Using the ProImmune REVEAL^®^ MHC-peptide binding assay, we assessed the binding of 95 overlapping 15-mer human CD20-derived peptides with an offset of 3 amino acids to recombinant human MHC II molecules frequently found in European populations (HLA-DRB1*01:01; HLA-DRB1*03:01; HLA-DRB1*04:01; HLA-DRB1*07:01). Six of these peptides failed in synthesis, and therefore could not be tested. The binding assays revealed frames of densely packed high-scoring peptides (Fig. 1a), and thus clusters of potentially immunogenic epitopes within the intracellular, transmembrane, and extracellular domains of the human CD20 molecule (Fig. 1b).

**Fig. 2:**
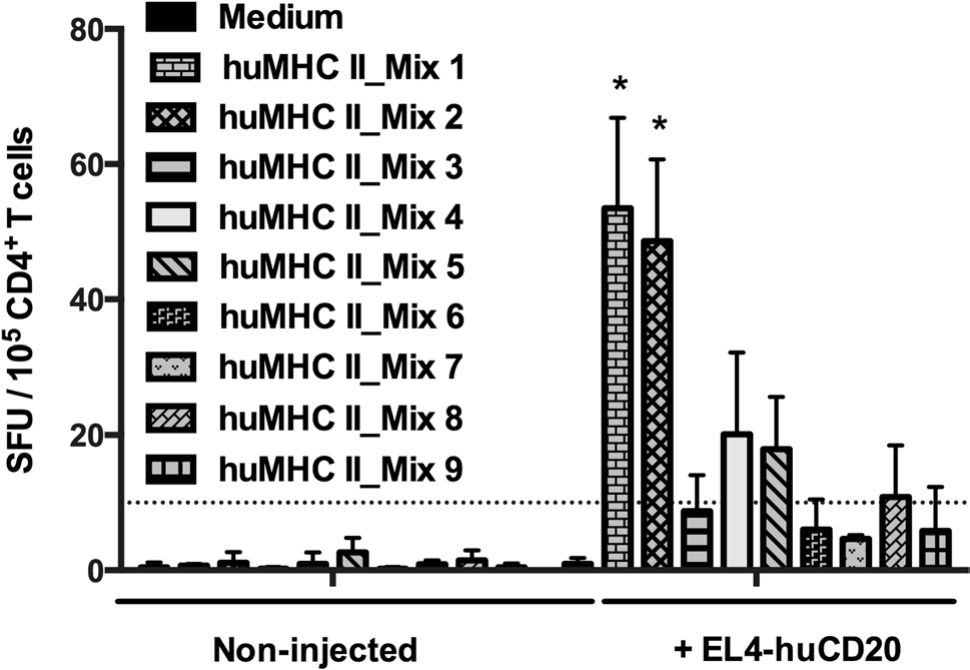
Induction of CD4^+^ T cell responses directed against human CD20-derived peptides in HLA-A2.1/HLA-DR1-transgenic H-2 class I-/class II-KO mice immunized with EL4-huCD20 tumor cells. The frequency of IFN-γ producing CD4^+^ T cells directed against CD20-derived peptides (huMHC II_Mix 1 to huMHC II_Mix 9) from HLA-A2.1/HLA-DR1-transgenic mice inoculated with EL4-huCD20 tumor cells (+ EL4-huCD20) or from their naive counterparts (non-injected) was evaluated by ELISPOT assays. Results were expressed as SFU per 10^5^ CD4^+^ T cells. Bars represent the mean values from two (huMHC II_Mix 3, huMHC II_Mix 5, huMHC II_Mix 6, huMHC II_Mix 7, huMHC II_Mix 8) or three (huMHC II_Mix 1, huMHC II_Mix 2, huMHC II_Mix 4, huMHC II_Mix 9) independent experiments. The positive threshold (horizontal dotted line) was set at ≥ 10 SFU per 10^5^ cells as previously described [30]. *Indicates that IFN-γ responses obtained with CD4^+^ T cells stimulated with huMHC II_Mix 1 or huMHC II_Mix 2 were significantly higher than those obtained in all other conditions (Multiple *t* tests followed by Bonferroni correction)

We then assessed whether peptides corresponding to these clusters defined in vitro might be presented by MHC molecules and promote T cell responses in vivo. H-2 KO/HLA-A2^+^-DR1^+^ transgenic mice were i.v. injected with 2 × 10^5^ tumor cells expressing the entire human CD20 molecule (EL4-huCD20). 21 days later, splenic CD4^+^ T cells were isolated and co-cultured in ELISPOT plates for 36 h with NIH/3T3 cell-derived AAPCs. These cells that express human HLA-DRB1*01:01 molecules loaded with 9 different mixtures of 18 to 20-mer MHCII-restricted peptides selected for their different high-scoring frames (huMHC II_Mix 1 to 9) (Fig. 1; Supplementary Table 1). ELISPOT assays showed that five mixtures of MHC II-restricted huCD20-derived peptides (huMHC II_Mix 1, huMHC II_Mix 2, huMHC II_Mix 4, huMHC II_Mix 5, and huMHC II_Mix 8) were able to stimulate IFN-γ production by CD4^+^ T cells isolated from mice injected with EL4-huCD20 tumor cells (Fig. 2). The responses induced by peptides of huMHC II_Mix 1 and of huMHC II_Mix 2 (localized in one of the intracellular domains of the CD20 molecule) were significantly higher as compared to the other mixtures (Fig. 2).

### MHC II-restricted CD20-derived peptides induce in vitro IFN-γ production by T cells from healthy individuals and follicular lymphoma patients

We then investigated whether HLA-DR-restricted CD20-derived peptides could stimulate the production of IFN-γ by PBMCs from healthy donors. Since a role for T cells bearing low-avidity TCRs for self-antigens in the immune surveillance of spontaneous spleen B cell lymphoma has been reported [31], we also assessed the IFN-γ production in response to these peptides using human spleen cells.

**Fig. 3:**
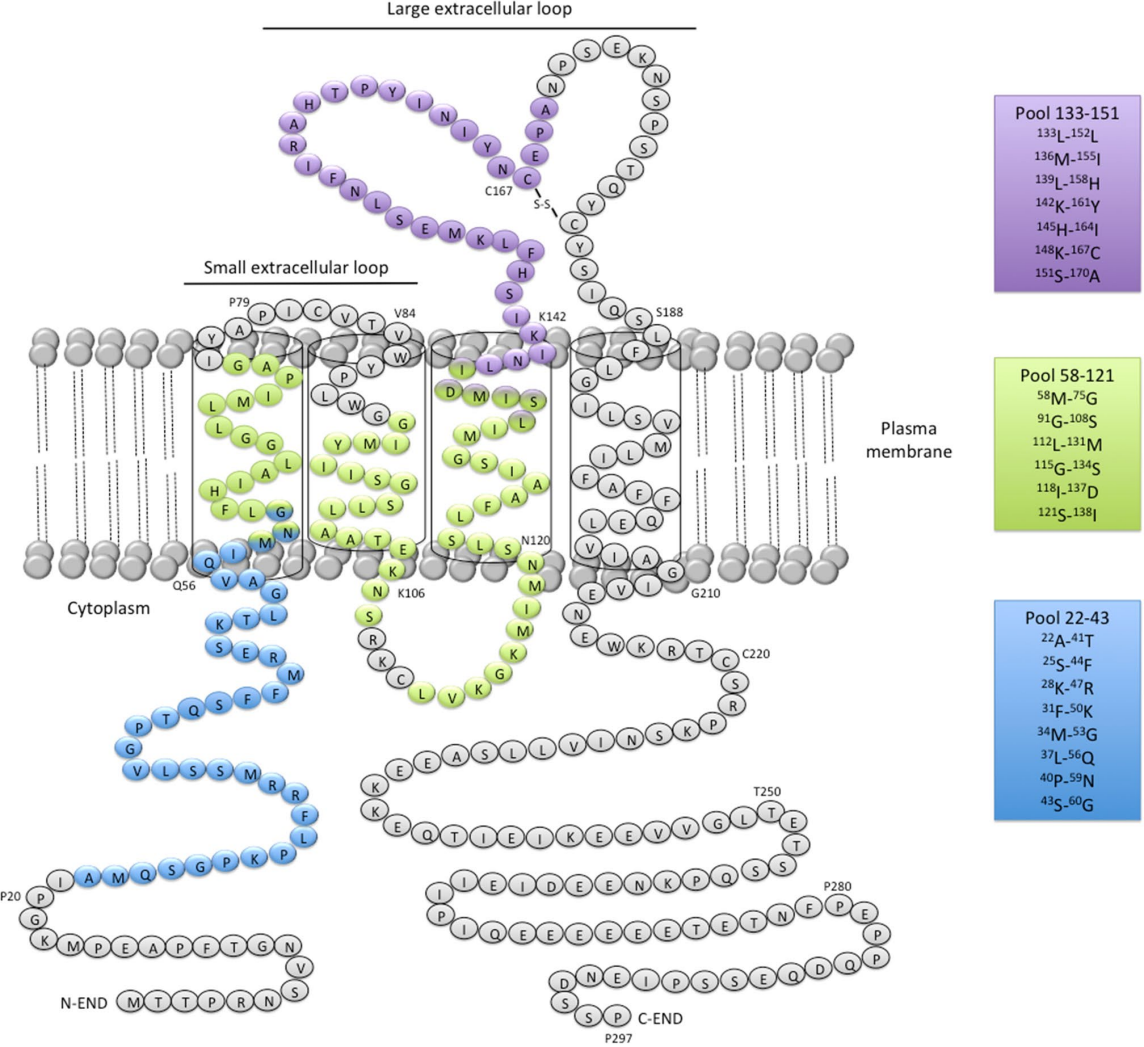
Localization of HLA-DR-restricted CD20-derived peptide pools used to analyze anti-CD20 T cell responses in human PBMCs and splenocytes. Pool 22–43 spans ^22^A-^60^G in blue, Pool 58–121, ^58^M-^138^I in green; Pool 133–151, ^133^L-^170^A in purple

**Fig. 4:**
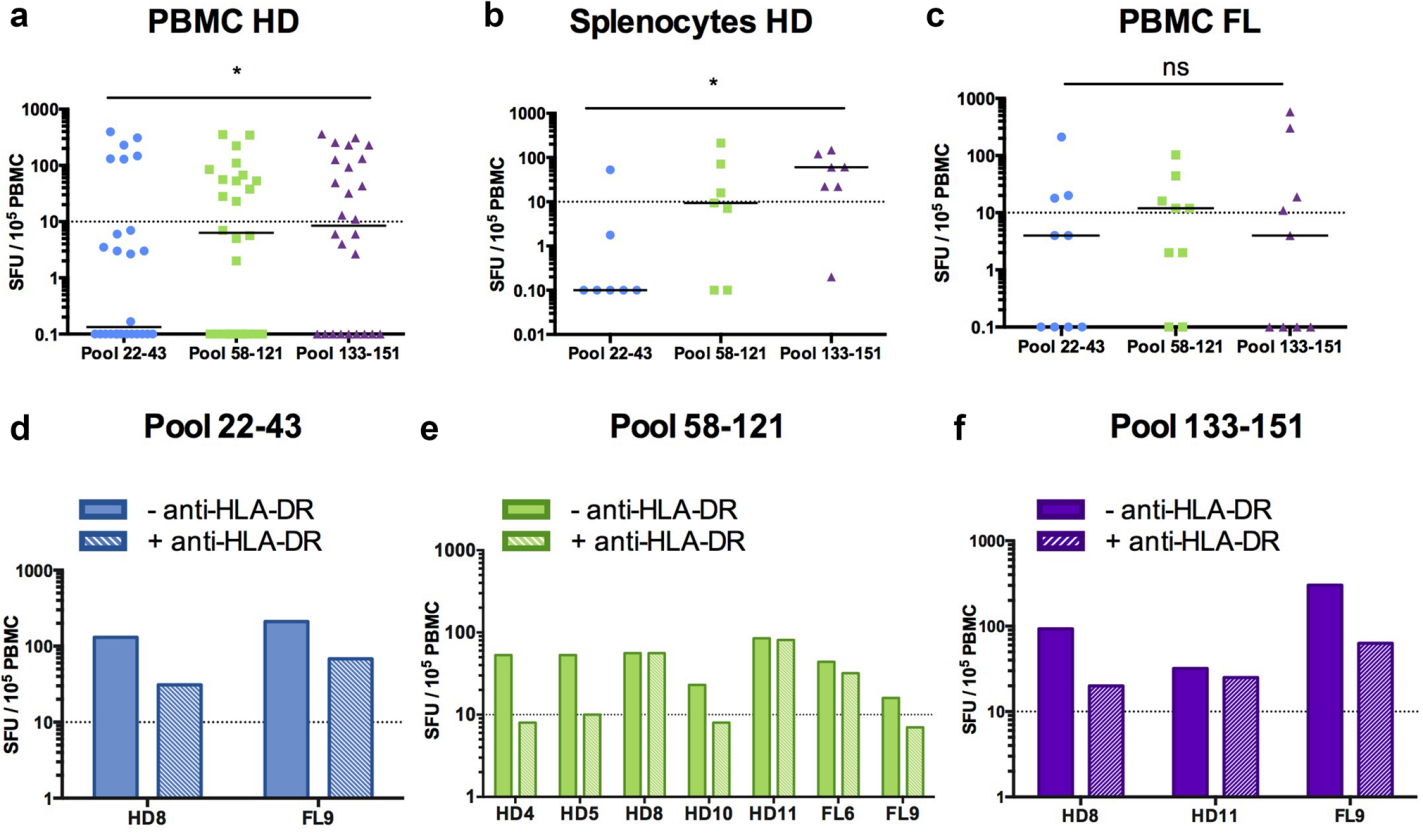
IFN-γ responses induced by HLA-DR-restricted human CD20-derived peptides in healthy individuals and follicular lymphoma patients. PBMCs from **a** healthy donors (PBMC HD, *n *= 26) or **c** follicular lymphoma patients (PBMC FL, *n *= 9), and **b** splenocytes of healthy individuals (splenocytes HD, *n *= 7) were incubated with pools of MHC II-restricted CD20-derived peptides (Pools 22–43, in blue; Pool 58–121, in green; Pool 133–151, in purple). IFN-γ production was then measured by ELISPOT assays as described in the “Materials and methods” section. Results were expressed as SFU per 10^5^ cells. Median values are indicated for each pool of peptides. The positive threshold (horizontal dotted line) was set at ≥ 10 SFU per 10^5^ cells as previously described [30]. Non-parametric paired Wilcoxon tests were used for statistical analysis. *p < 0.05; *ns* not significant. **d**–**f** IFN-γ production by PBMCs from healthy donors (HD4, HD5, HD8, HD10, HD11) or from FL patients (FL6 and FL9) measured by ELISPOT assays in response to **d** Pool 22–43, **e** Pool 58–121 or **f** Pool 133–151, in absence (− anti-HLA-DR) or presence (+ anti-HLA-DR) of blocking anti-HLA-DR, -DP, -DQ monoclonal antibody

Three pools comprising all the peptides that activated CD4^+^ T cells in H-2 KO/HLA-A2^+^-DR1^+^ transgenic mice were designed for human ELISPOT assays (Fig. 3). The first one (termed pool 22–43) contained all the peptides of huMHC II_Mix. 1, huMHC II_Mix. 2, and huMHC II_Mix. 3. The pool 58–121 included huMHCII_Mix. 4 and huMHCII_Mix. 5 peptides, and the last one, termed 133–151, was obtained by pooling huMHC II_Mix. 6, huMHC II_Mix. 7, and huMHCII_Mix. 8 peptides (Fig. 3; Supplementary Table 1). All three pools of MHC II-restricted CD20-derived peptides induced T cell responses by PBMCs or spleen cells from a number of healthy individuals (Fig. 4a, b; Supplementary Table 2). The median SFU value per 10^5^ cells was significantly higher for pool 133–151 (peptides located within the large extracellular loop of CD20) as compared to pool 22–43 (peptides located within the intracellular domain) (Fig. 4a, b). This was observed both with PBMCs and splenocytes. Various response profiles were observed among the HD PBMC samples. 23% exhibited IFN-γ production in response to each of the three pools of peptides. Responses to one or two out of the three pools were detected in 19% and 15% of HD PBMC samples, respectively. IFN-γ production in response to one or two of the three pools was detected in 28% or 57% of splenocytes, respectively (Supplementary Fig. 1). Of note, FVIII peptides used as a control for self-antigen-derived peptides (see “Materials and methods”) did not induce IFN-γ production in PBMCs from healthy donors (data not shown).

Finally, we assessed whether CD20-specific T cell responses could be detected in PBMCs from patients diagnosed with high tumor-burden FL and treated with R-CHOP (Fig. 4c). Again, the IFN-γ ELISPOT assays revealed T cell responses against all the peptide pools (Supplementary Table 2). No significant differences in median SFU per 10^5^ cells were detected between the different pools of peptides (Fig. 4c), and about the same percentages of responses to one or three pools were observed (Supplementary Fig. 1). The intensity of IFN-γ response detected in ELISPOT assays with the three pools of peptides was similar between the different types of samples (PBMCs and splenocytes from healthy individuals, and PBMCs from FL patients) (Supplementary Fig. 2).

To further analyze the involvement of CD4^+^ T cells in IFN-γ responses detected in ELISPOT assays, a monoclonal anti-HLA-DR, -DP, -DQ blocking antibody was added to culture of PBMCs from several healthy donors and from FL patients in the presence of the different pools of CD20-derived peptides (Fig. 4d–f). The IFN-γ production could be blocked by the anti-HLA-DR, -DP, -DQ antibody for some, but not all the samples tested (Fig. 4d–f and Supplementary Fig. 3). Thus, these results indicate that CD4^+^ T cells are implicated in IFN-γ response and also suggest that the presence of CD20-derived long peptides could stimulate CD8^+^ T cells.

**Fig. 5:**
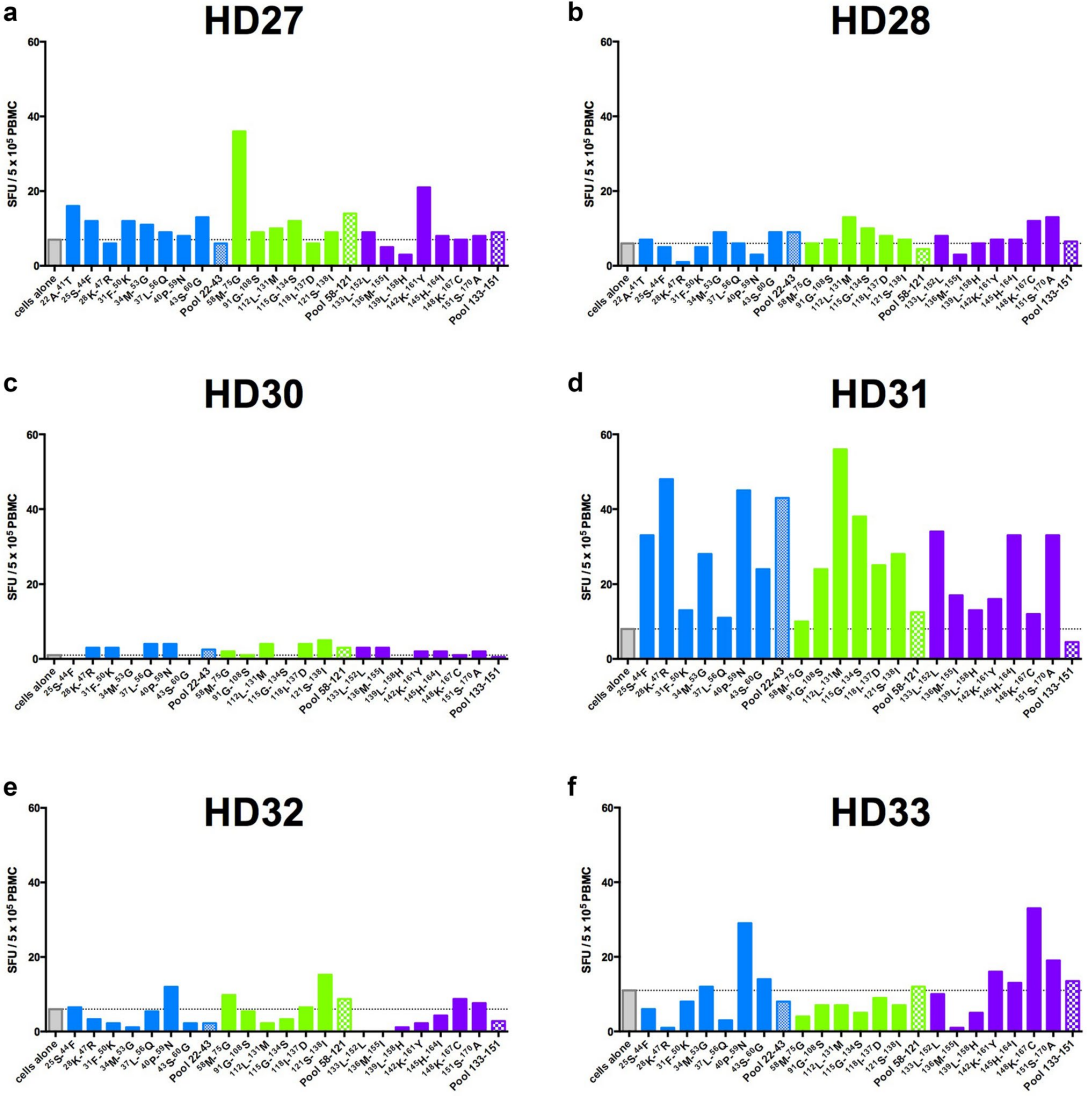
IFN-γ responses to individual peptides by PBMCs from healthy donors HD27 (**a**), HD28 (**b**), HD30 (**c**), HD31 (**d**), HD32 (**e**), HD33 (**f**). Horizontal dotted line and gray bar represent the number of SFU/5 × 10^5^ PBMCs obtained when cells are cultured alone (background). Bars represent results from individual peptides from pool 22–43 (blue), pool 58–121 (green), and pool 133–151 (purple) (one well/peptide). Hatched bars represent results with the three pools: pool 22–43, blue; pool 58–121, green; pool 133–151, purple (1 well/pool)

All the ELISPOT assays were performed after expanding cells in vitro for 7 days in presence of IL-2, IL-7, and peptide pools. Both naive T cell priming and memory-specific T cell expansion can occur in this setting. To analyze the memory T cell pool specifically, we performed ex vivo ELISPOT assays with the pools of peptides or with individual peptide incubated with 5 × 10^5^ PBMCs for 48 h in absence of cytokines. A low background was observed in 6/10 of the HD samples tested when cells were cultured without peptide. HD31 exhibited a marked response to pool 22–43 whereas responses to the other peptide pools were barely detected in these 6 donors (Fig. 5). Interestingly, when single peptides were tested, responses above the baseline could be observed in HD27 (^58^M-^75^G; ^142^K-^161^Y), HD31 (^25^S-^44^F; ^28^K-^47^R; ^34^M-^53^G; ^40^P-^59^N; ^43^S-^60^G; ^91^G-^108^S; ^112^L-^131^M; ^115^G-^134^S; ^118^I-^137^D; ^121^S-^138^I; ^133^L-^152^L; ^145^H-^164^I; ^151^S-^170^A), and HD33 (^40^P-^59^N; ^148^K-^167^C). Thus, memory T cells against CD20-derived peptides can be detected in some healthy donors using a short-term in vitro incubation.

Taken together, these results show that T cell responses against MHC II-restricted CD20-derived peptides are detected in samples from healthy donors and FL patients. While these T cells recognize epitopes located in different domains of the CD20 protein (extracellular, transmembrane, and intracellular domains), the intensity and frequency of T cell responses against epitopes in the large extracellular loop appear to be higher, at least in healthy individuals.

## Discussion

Our previous studies performed in mice bearing EL4-huCD20 tumor cells have demonstrated that a protective CD4^+^ T cell response directed against human CD20 molecule is induced after mAb treatment [12, 13]. However, the relevant MHC II-restricted T cell epitopes are unknown. Thus, we investigated herein the presence of T cell epitopes in human CD20 and whether T cells directed against CD20-derived peptides can be detected in human PBMCs and splenocytes.

Based on in vitro binding assays to recombinant human MHC II molecules (frequent alleles in European populations, i.e., HLA-DR1; HLA-DR3; HLA-DR4; HLA-DR7) and on in vivo immunization of H-2 KO/HLA-A2^+^-DR1^+^ transgenic mice, we have identified three pools of human MHC II-restricted T cell peptides located in different domains of the CD20 protein that induce in vitro IFN-γ responses in samples from healthy donors and FL patients (Fig. 4). Of note, some differences were observed between H-2 KO/HLA-A2^+^-DR1^+^ transgenic mice immunization and in vitro tests of human PBMCs. In experiments using H-2 KO/HLA-A2^+^-DR1^+^ transgenic mice, the responses induced by peptides localized in the N-terminal intracellular domain of CD20 molecule (huMHC II_Mix 1 and huMHC II_Mix 2, position 22–56) were significantly higher as compared to the other peptides (Fig. 2). By contrast, when both human PBMCs and splenocytes were tested in vitro, the median SFU value per 10^5^ cells was significantly higher for pool 133–151 (peptides located within the large extracellular loop of CD20) as compared to the other pools (Fig. 4). These differences could be due to the fact that responses achieved in H-2 KO/HLA-A2^+^-DR1^+^ transgenic mice result from the presentation of CD20-derived peptides solely by an HLA-DR1 molecule. By contrast, the PBMCs used in ELISPOT assays are derived from individuals with HLA-DR1 and/or HLA-DR3, HLA-DR4, HLA-DR7 haplotypes. The use of H-2 KO/HLA-A2^+^-DR1^+^ transgenic mice inoculated with EL4-huCD20 tumor cells enables the detection of mouse T cell responses directed against human CD20, a xenogeneic antigen in this setting, in contrast to the assays with human samples in which autologous CD20-derived peptides are used. Nevertheless, this preclinical model represents a valuable tool to establish that peptides selected in silico can be presented in vivo by human HLA-DR1.

Our results also indicate that peptides derived from the huCD20 sequence ^133^L-^170^A (located in the large extracellular loop) are the most immunogenic. This observation is reminiscent of a previous study showing the induction of an antibody response in BALB/c mice vaccinated with a peptide from the human CD20 extracellular loop sequence (CKISHFLKMESLNFIRAHTPYINIYNCEPANPSEKNS PSTQYCY) [32]. However, although the intensity and frequency of T cell responses against epitopes localized in the large extracellular loop appear to be higher, at least in healthy donors, IFN-γ responses to peptides derived from the intracellular and transmembrane domains (Pools 22–43 and 58–121) were also detected in some individuals. Thus, in addition to MHC I-restricted peptides derived from the extracellular CD20 loop previously described [16–22], we were able herein to define 15 to 20-mer MHC II-restricted T cell epitopes derived from either intracellular, membrane, or extracellular domains of the human non-mutated CD20 protein. Of note, the data obtained in the presence of anti-HLA-DR, -DP, -DQ blocking antibody suggest that these long peptides also have the ability to stimulate CD8^+^ T cells in vitro for some individuals as already reported in other studies [reviewed in 33].

We cannot exclude that the immunogenic peptides for which a CD4^+^ T cell response is detected in individuals included in our study are different to the endogenously expressed CD20 polypeptide. Low-frequency mutations including SNPs or polymorphisms of the CD20-encoding *MS4A1* gene have been observed in NHL patients [34–37]. It has been suggested that some CD20 tumor-associated mutations could be treatment induced [37]. Five CD20 alternative splice variants have also been identified in human Epstein–Barr Virus (EBV)-transformed B cell lines and in primary samples of FL, CLL, mantle cell lymphoma (MCL) or diffuse large B cell lymphoma (DLBCL) patients [38–40]. Interestingly, specific T cell responses against a 20-mer peptide derived from one of these CD20 splice variants (D393-CD20) were detected in both lymphoma patients and healthy individuals [39]. However, it is important to stress that no splice variants were observed in normal B cells from healthy donors in these studies and that the different splice variants and the wild-type CD20 isoform are co-expressed in NHL B cell patients [39, 40]. It is thus unlikely that an allogeneic T cell response is being observed rather than an autoreactive T cell response in our experimental setting.

Non-mutated self-proteins overexpressed on tumor cells are a source of universal target antigens for inducing tumor-specific T lymphocytes without the need to identify the mutanome of tumor cells. Recent results have demonstrated that thymic deletion prunes but does not eliminate self-specific CD4^+^ and CD8^+^ T cells, and that some self-peptide/MHC-restricted T cells can be detected at frequencies similar to those of T cells specific for non-self-antigens [41–44]. While the use of such epitopes could be limited by self-tolerant T cell repertoire, therapeutic strategies have been developed to overcome the tolerance of T cells to self-peptides. For example, adjuvants, lentivectors, or inhibitory immune checkpoint blocking molecules can improve the efficacy of self-peptide-based vaccinations [45, 46]. Moreover, anti-CA125, anti-HER2/neu, anti-MUC1, anti-EGFR mAb treatment can circumvent the tolerance to self-antigens expressed on tumor cells as shown by the increase of the frequency of CD4^+^ and/or CD8^+^ T cells recognizing peptides derived from the target molecule in cancer patients [47–51].

In our experimental setting, priming of naive T cells in addition to the activation of memory T cells can likely occur during the 7-day expansion. Different studies have shown that T cells specific to a given antigen can be detected in the naive but not in the memory T cell compartment in non-immune donors [52, 53]. This is consistent with the high diversity of the naive repertoire as compared to the much lower diversity of the memory repertoire, which represents a collection of clones selected during immune responses. In these studies, an amplification step has been used to detect these specific T cells due to their very low frequencies in the naive repertoire. These observations underline the importance of exploring both the naive and memory repertoires to identify anti-CD20-specific CD4^+^ T cells that can be manipulated in the context of vaccination strategies. Our data suggest that both naive and memory anti-CD20 T cells can be present in healthy donors.

In conclusion, our results indicate that carefully selected CD20-derived MHC II-restricted peptides make it possible to induce CD20-specific CD4^+^ T cell responses in humanized HLA-DR-transgenic mice and in human PBMCs. These peptides could serve as a therapeutic tool in B cell malignancies to improve the antitumor activity of CD4^+^ T cells in the context of vaccination strategies by helping CD8^+^ T cell response and eventually through direct cytotoxic effector functions [54]. Furthermore, our results indicate that anti-CD20 T cells present in FL patients exhibit various epitope specificities (Fig. 2; Supplementary Table 2). This finding suggests that any vaccination approach based on the use of CD20-derived peptide pools should include pre-screening of patients who respond to these pools. CD20-derived peptides could also be used ex vivo to develop an adoptive T cell immunotherapy strategy. Finally, they could help in monitoring the anti-tumor T cell responses in patients treated with rituximab or other anti-CD20 antibodies.

## Electronic supplementary material

Below is the link to the electronic supplementary material.

Supplementary material 1 (PDF 255 kb)

## Abbreviations

AAPC: Artificial antigen-presenting cells
CTL: Cellular Technology Limited
DLBCL: Diffuse large B cell lymphoma
EFS: Etablissement Français du Sang
FVIII: Factor VIII
FL: Follicular lymphoma
HD: Healthy donor
KO: Knock-out
MCL: Mantle cell lymphoma
R-CHOP: Rituximab, cyclophosphamide, doxorubicin, vincristine, prednisolone
SFU: Spot forming units
