# Supplementary material for: Presence of T cells directed against CD20-derived peptides in healthy individuals and lymphoma patients
Source: Cancer Immunol Immunother. 2019 Sep 7;68(10):1561–72. doi: 10.1007/s00262-019-02389-7 (PMC6805815; doi:10.1007/s00262-019-02389-7)
Supplement: Supplementary file 1 — Supplementary material 1 (PDF 255 kb) [file 262_2019_2389_MOESM1_ESM.pdf]

Supplementary Table 1. Sequences of HLA-DR-restricted CD20-derived peptides

Peptide Sequence	Number of amino-acids
**huMHC II_Mix 1**	
^{22}AMQSGPKPLFRRMSSLVGPT^{41}	20
^{25}SGPKPLFRRMSSLVGPTQSF^{44}	20
^{28}KPLFRRMSSLVGPTQSFFMR^{47}	20
**huMHC II_Mix 2**	
^{31}FRRMSSLVGPTQSFFMRESK^{50}	20
^{34}MSSLVGPTQSFFMRESKTLG^{53}	20
^{37}LVGPTQSFFMRESKTLGAVQ^{56}	20
**huMHC II_Mix 3**	
^{40}PTQSFFMRESKTLGAVQIMN^{59}	20
^{43}SFFMRESKTLGAVQIMNG^{60}	18
**huMHC II_Mix 4**	
^{58}MNGLFHIALGGLLMIPAG^{75}	18
^{91}GIMYIISGSLLAATEKNS^{108}	18
**huMHC II_Mix 5**	
^{112}LVKGKMIMNSLSLFAAISGM^{131}	20
^{115}GKMIMNSLSLFAAISGMILS^{134}	20
^{118}IMNSLSLFAAISGMILSIMD^{137}	20
^{121}SLSLFAAISGMILSIMDI^{138}	18
**huMHC II_Mix 6**	
^{133}LSIMDILNIKISHFLKMESL^{152}	20
^{136}MDILNIKISHFLKMESLNFI^{155}	20
^{139}LNIKISHFLKMESLNFIRAH^{158}	20
**huMHC II_Mix 7**	
^{142}KISHFLKMESLNFIRAHTPY^{161}	20
^{145}HFLKMESLNFIRAHTPYINI^{164}	20
**huMHC II_Mix 8**	
^{148}KMESLNFIRAHTPYINIYNC^{167}	20
^{151}SLNFIRAHTPYINIYNCEPA^{170}	20
**huMHC II_Mix 9**	
^{175}KNSPSTQYCYSIQSLFLG^{192}	18
^{214}NEWKRTCSRPKSNIV^{228}	15

Supplementary Table 2. IFN-γ responses to CD20-derived human MHC-restricted peptides in cells from healthy donors (PBMCs, *n*=26; splenocytes, *n*=7) and FL patients (PBMCs) (*n*=9)

Samples	Pool 22-43* (SFU/10^5 cells)	Pool 58-121* (SFU/10^5 cells)	Pool 133-151* (SFU/10^5 cells)
PBMC HD1	0	0	6
PBMC HD2	0	0	0
PBMC HD3	0	0	0
PBMC HD4	0	53	4
PBMC HD5	3	53	11
PBMC HD6	3	28	43
PBMC HD7	7	7	13
PBMC HD8	131	56	93
PBMC HD9	0	0	0
PBMC HD10	0	23	6
PBMC HD11	6	85	32
PBMC HD12	309	352	309
PBMC HD13	397	346	361
PBMC HD14	147	109	126
PBMC HD15	229	223	255
PBMC HD16	0	0	0
PBMC HD17	0	0	0
PBMC HD18	0	0	0
PBMC HD19	0	0	0
PBMC HD20	0	2	0
PBMC HD21	3	5	49
PBMC HD22	129	67	230
PBMC HD23	2	5	2
PBMC HD24	0	37	230
PBMC HD25	0	0	131
PBMC HD26	0	0	0
Splenocytes 1	0	212	60
Splenocytes 2	0	9	22
Splenocytes 3	0	7	22
Splenocytes 4	0	0	0
Splenocytes 5	1	16	60
Splenocytes 6	0	71	147
Splenocytes 7	53	0	120

PBMC FL1	18	103	581
PBMC FL2	4	2	11
PBMC FL3	20	12	19
PBMC FL4	0	0	0
PBMC FL5	0	12	0
PBMC FL6	4	44	4
PBMC FL7	0	2	0
PBMC FL8	0	0	0
PBMC FL9	211	16	302

*IFN-γ production in response to human CD20-derived peptide mixture was analyzed by ELISPOT assays as described in the Materials and Methods section. Numbers represent the mean value of triplicates. The positive threshold was set at ≥ 10 spot forming units (SFU) per 10^5 cells as previously described [30]. HD: healthy donors.

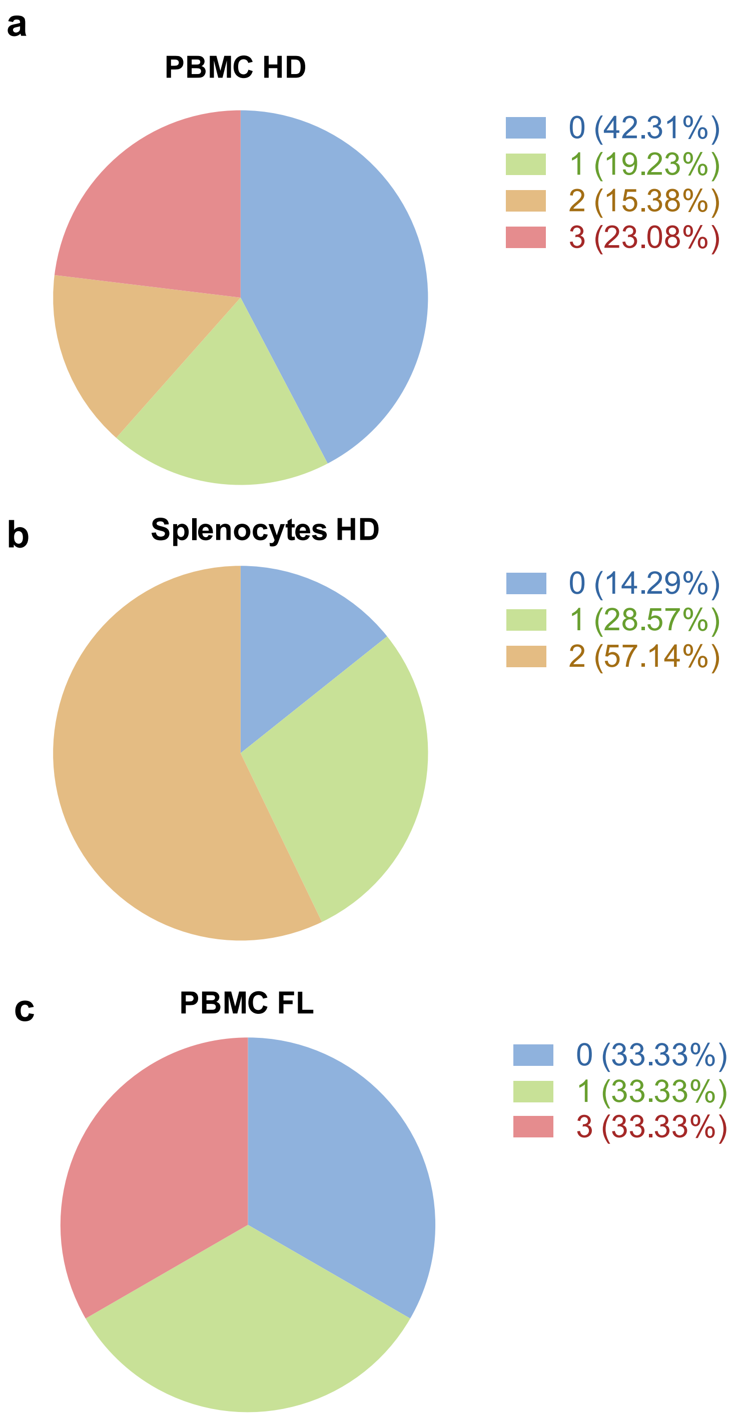

Supplementary Figure 1. Frequency of non-responders or responders to one or several pools of MHC II-restricted CD20-derived peptides among healthy individuals or FL patients. Pie charts indicate the percentages of IFN-γ responses by PBMC from healthy donors (PBMC HD, *n*=26) **(a)**, by splenocytes from donors (splenocytes HD, *n*=7) **(b)** or by PBMCs from follicular lymphoma patients (PBMC FL, *n*=9) **(c)** to one (in green), two (in orange), or three (in red) pools of CD20-derived peptides. The percentages of non-responders are indicated in blue.

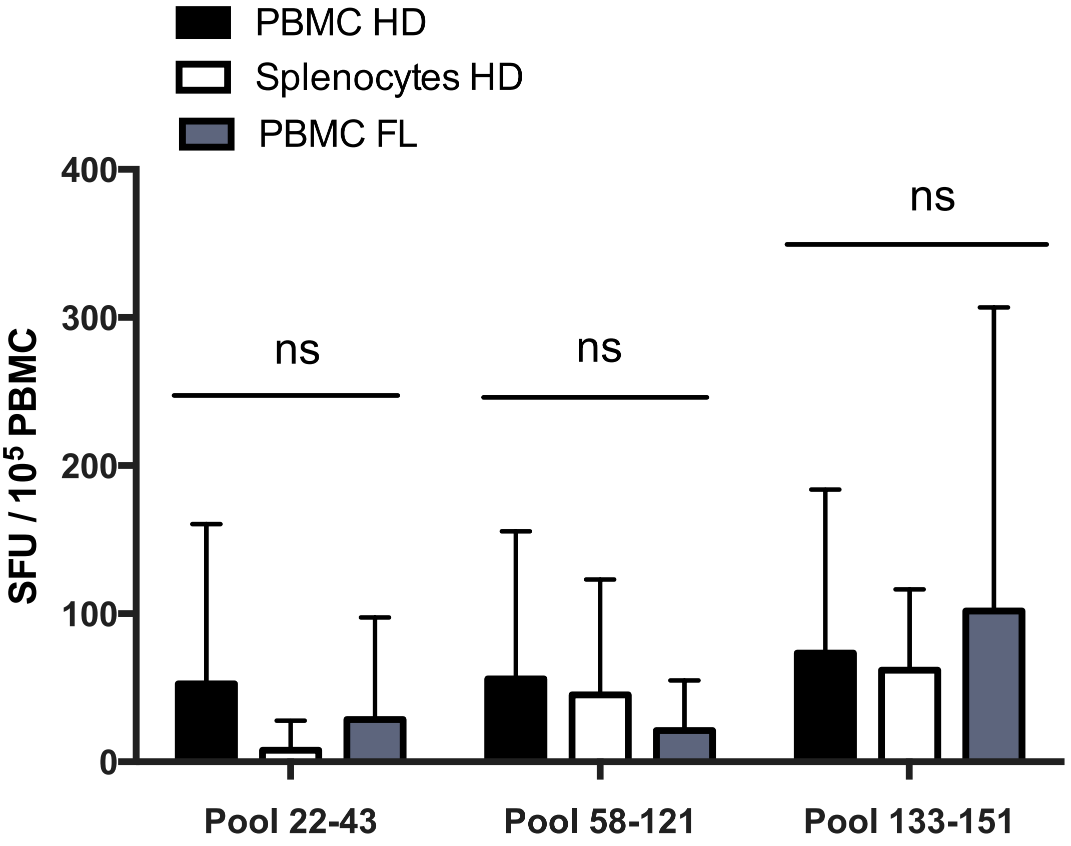

Supplementary Figure 2. Comparison of IFN-γ responses to the different pools of MHC II-restricted CD20-derived peptides (Pool 22-43, Pool 58-121, Pool 133-151) in PBMCs from healthy donors (PBMC HD, *n*=26, black bars) or from follicular lymphoma patients (PBMC FL, *n*=9, grey bars), and in splenocytes from healthy individuals (splenocytes HD, *n*=7, white bars). IFN-γ production was measured by ELISPOT assays as described in the materials and methods section. Results were expressed as mean value of spot-forming units (SFU) per 10^5 cells. Nonparametric unpaired Mann-Whitney tests were used for statistical analysis. ns: not significant.

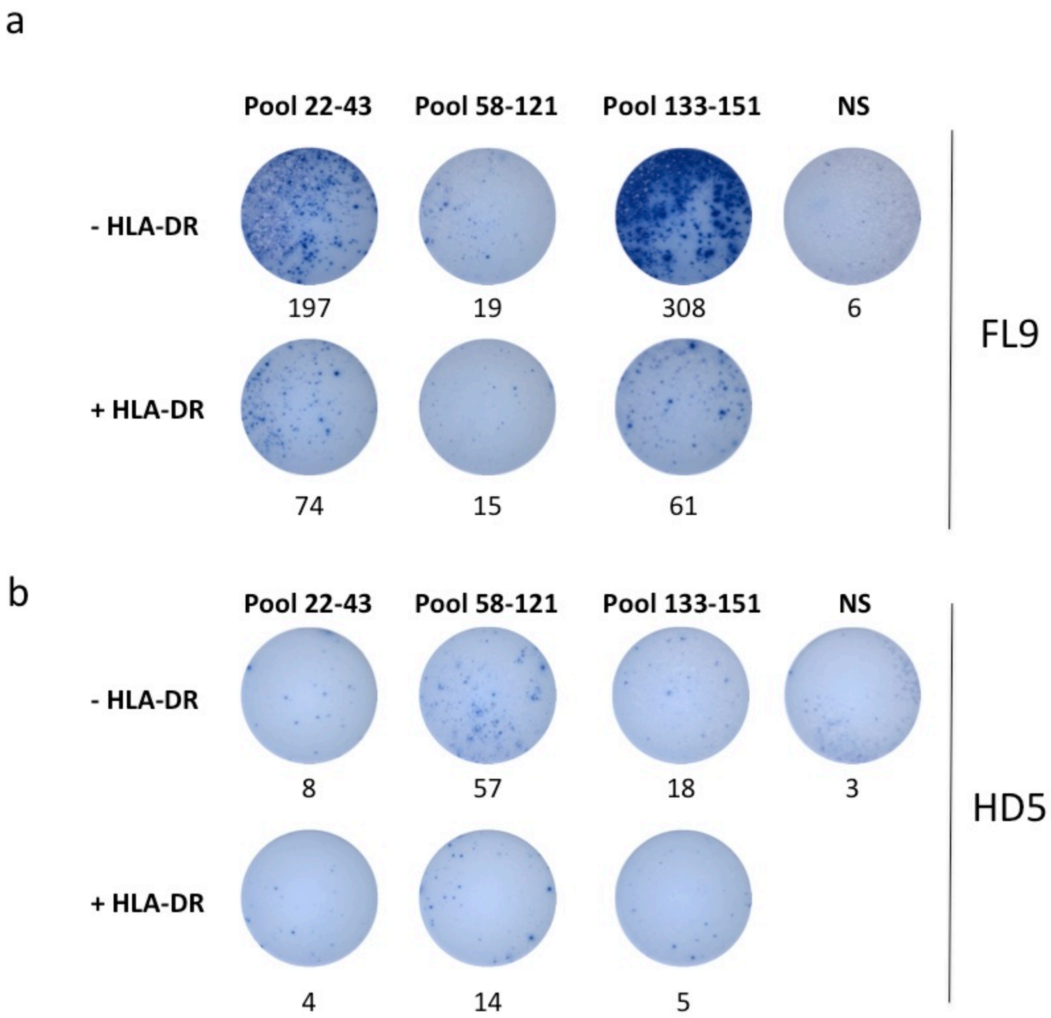

Supplementary Figure 3. Representative images of ELISPOT wells depicting IFN-γ responses by PBMCs from one FL patient (FL9) **(a)** and one HD (HD5) **(b)** and their blockade by an anti-HLA-DR, -DP, -DQ antibody. Numbers below images indicate the number of SFU/10^5 cells obtained in the corresponding well.

[1] NS: non-stimulated cells.
